# New advances in the treatment of chronic rhinosinusitis with nasal polyps from pathophysiology to biologic targeted therapy

**DOI:** 10.3389/falgy.2026.1903649

**Published:** 2026-09-11

**Authors:** Baozhu Huang, Yue Xu, Ye Tian, Xiaoyu Liu, Xiaoyu Gao, Xue Wang, Yurong Zhang, Xiaoman Li, Ting Liu, Weibang Chen, Wenya Gao, Huikuan Chen, Feng Li, Danxian Jiang, Junfeng Hao, Chunjie Tian

**Affiliations:** 1 Department of Otorhinolaryngology, Affiliated Hospital of Guangdong Medical University, Zhanjiang, Guangdong, China; 2 Maoming People’s Hospital, Maoming, Guangdong, China; 3 Head and Neck Oncology Specialty, Affiliated Hospital of Guangdong Medical University, Zhanjiang, China; 4 Guangdong Medical University, Zhanjiang, China; 5 Guangdong Provincial Key Laboratory of Autophagy and Major Chronic Non-communicable Diseases, Zhanjiang, China; 6 Guangdong Provincial Key Laboratory of Autophagy and Major Chronic Non-communicable Diseases, Institute of Nephrology, Affiliated Hospital of Guangdong Medical University, Zhanjiang, Guangdong, China; 7 Department of Family Medicine, Shengjing Hospital of China Medical University, Shenyang, Liaoning, China

**Keywords:** biologic therapy, chronic rhinosinusitis with nasal polyps (CRSwNP), eosinophils, interleukins, precision medicine, type 2 inflammation

## Abstract

**Background:**

Chronic rhinosinusitis with nasal polyps (CRSwNP) is a chronic airway disease characterized mainly by type 2 inflammation, which seriously impairs patients' quality of life and imposes a huge economic burden. Traditional treatments cannot eradicate underlying immune disorders, leading to high postoperative recurrence. This review aims to summarize the pathophysiological basis of CRSwNP and the clinical application advances of biologics in its precision treatment.

**Methods:**

A narrative review was performed to synthesize key literature on the pathophysiology of CRSwNP, focusing on the core role of type 2 inflammatory molecules (IL-4, IL-13, IL-5, IgE, TSLP) and the clinical evidence of targeted biologics for refractory CRSwNP.

**Results:**

In-depth understanding of CRSwNP pathophysiology has promoted the shift to precision medicine. Biologics including dupilumab (anti-IL-4Rα), mepolizumab (anti-IL-5), omalizumab (anti-IgE) and tezepelumab (anti-TSLP) can precisely target key molecules in type 2 inflammatory pathways, achieving effective and long-lasting disease control, reducing polyp volume, improving nasal symptoms and olfactory function, decreasing reliance on oral corticosteroids and the need for reoperation, and exerting synergistic effects on comorbid asthma.

**Conclusions:**

Biologics mark a milestone in CRSwNP treatment, shifting clinical management from symptomatic treatment to individualized decision-making based on patient endotypes, comorbidities and treatment goals such as disease remission. Future research will focus on predictive biomarkers, head-to-head comparative studies and new targets for non-type 2 inflammation to optimize long-term management. By integrating recent biologic evidence with endotype-based recurrence mechanisms, this review provides a practical framework for biologic stewardship and remission-oriented management in CRSwNP.

## 1 Introduction

### 1.1 Definition, epidemiology, and disease burden of CRSwNP

Chronic Rhinosinusitis (CRS) is a complex disease characterized by persistent inflammation of the nasal and paranasal sinus mucosa, clinically defined by the presence of relevant symptoms (such as nasal congestion, rhinorrhea, facial pain/pressure, and reduced sense of smell) for 12 weeks or more, accompanied by objective evidence from nasal endoscopy or imaging studies (1, 2). Chronic Rhinosinusitis with Nasal Polyps (CRSwNP) is a distinct subtype, characterized by the formation of grape-like benign growths within the nasal cavity and sinuses (3).

The multi-dimensional burden of CRSwNP can severely impair health-related quality of life (HRQoL) across physical and psychological domains (4). Primarily, cardinal symptoms—including persistent nasal obstruction, purulent rhinorrhea, and anosmia—largely leading to chronic fatigue and cognitive dysfunction (5, 6). Furthermore, the chronic recurrent and treatment-refractory nature of the disease contributes to a high prevalence of comorbid anxiety and depression (7). Ultimately, this bidirectional physical and psychological burden compromises social functioning and raises indirect socioeconomic costs by increasing work presenteeism and absenteeism among patients (8). In addition, CRSwNP often coexists with lower airway diseases, particularly asthma, reflecting the concept of “one airway, one disease” (9, 10). Patients with CRSwNP and comorbid asthma typically tend to have more severe disease, greater treatment difficulties, and poorer prognosis,necessitating the recognition of this disease from the perspective of systemic inflammation.

### 1.2 The paradigm shift from traditional treatment to precision medicine

Traditional CRSwNP treatment adopts a stepwise approach: medical therapy based on intranasal corticosteroids, followed by endoscopic sinus surgery (FESS) if ineffective (11). Intranasal steroids aim to control local inflammation, while the goal of FESS is to remove polyps and widen the sinus ostia to improve drainage, thereby alleviating symptoms and enhancing the penetration of topical medications. However, this traditional model faces significant challenges. Most notably, even after thorough surgery, the recurrence rate of CRSwNP remains high, with some studies reporting a 5-year recurrence rate of up to 60% (12). Indicating that simply removing the diseased tissue (polyps) does not eradicate the underlying biological mechanisms driving the disease.

This has spurred a fundamental change in the management philosophy of CRSwNP (4, 13). The medical community has gradually recognized that CRSwNP is not a single anatomical problem but a heterogeneous group of diseases driven by different immunological pathways, i.e.,different “endotypes.” This deepening understanding has propelled the treatment strategy from a “one-size-fits-all” symptomatic approach to a “precision medicine” paradigm based on pathophysiological mechanisms (4, 13). The emergence of biologics in the form of monoclonal antibodies targeting specific inflammatory molecules or pathways is at the core of this revolution.

Although multiple reviews have summarized the efficacy and safety of individual biologics and pivotal trial data in CRSwNP, an integrated framework linking immunopathological mechanisms, inflammatory endotypes and clinical decision-making remains needed. The present review therefore provides a comprehensive and updated synthesis of current and emerging biologics within a mechanism-based disease framework. In addition to summarizing current and emerging targeted therapies, we integrate recent advances in type 2 and non-type 2 inflammatory pathways, including epithelial alarmins (TSLP, IL-33) and newly identified recurrence-associated mechanisms such as the GZMK^+^ CD8^+^ T cell–complement axis (14). By placing these data within an endotype-oriented clinical framework, postoperative recurrence can be interpreted not merely as surgical failure, but as a marker of persistent immune dysregulation that may guide earlier endotype-based biologic selection. This perspective may help position current and emerging biologics more precisely within individualized CRSwNP management and future drug development.

## 2 In-depth analysis of the pathophysiological mechanisms of CRSwNP

In recent years, with the advancement of molecular biology techniques, our understanding of the development of CRSwNP has deepened to the endotype level, with type 2 inflammation being identified as the core mechanism driving the majority of CRSwNP cases (especially in Western populations) (3, 15).

### 2.1 The central role and Key pathways of type 2 inflammation

Type 2 inflammation is an immune response dominated by type 2 helper T cells (Th2) and type 2 innate lymphoid cells (ILC2), characterized by eosinophilic infiltration and high expression of specific cytokines such as IL-4, IL-5, and IL-13.

#### 2.1.1 The IL-4/IL-13 pathway

**Figure 1:**
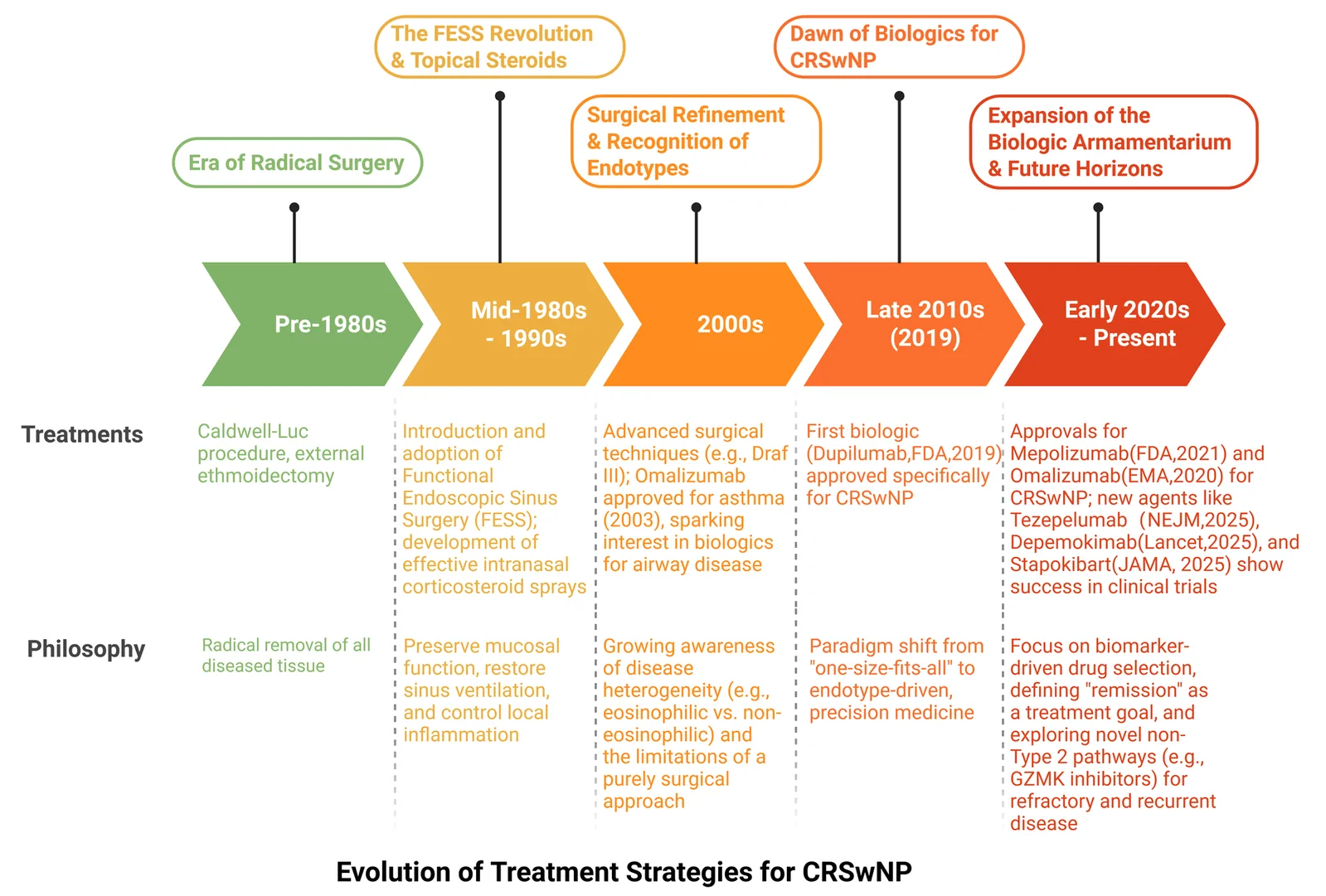
Timeline of CRSwNP treatment evolution. From radical surgery before the 1980s, through the introduction of functional endoscopic sinus surgery (FESS) and topical corticosteroids, to the recent rise of personalized therapy and biologics, this timeline illustrates key milestones in the evolution of treatment philosophy, surgical techniques, and pharmacotherapy for CRSwNP. The revised figure/legend highlights the shift from a one-size-fits-all approach toward precision medicine and biomarker-driven treatment strategies, and annotates major biologic milestones including dupilumab, omalizumab, mepolizumab, tezepelumab and emerging next-generation agents. Created with BioRender.com.

Interleukin-4 (IL-4) and Interleukin-13 (IL-13) are pivotal cytokines in the type 2 inflammatory response, exerting a wide range of biological effects through their shared IL-4Rα receptor chain. Research by Bachert et al. has detailed the central role of this pathway in CRSwNP (16). They not only drive B-cell class switching to IgE, promoting allergic reactions, but also act directly on airway epithelial cells to disrupt the integrity of the epithelial barrier, induce goblet cell hyperplasia, and promote mucus hypersecretion. Concurrently, they recruit and activate eosinophils, facilitating their infiltration into tissues. The tremendous clinical success of biologics targeting IL-4Rα (such as dupilumab) has, in turn, confirmed the critical role of the IL-4/IL-13 pathway in the pathogenesis of CRSwNP (17) (Figure 1).

#### 2.1.2 The IL-5 pathway

As a critical regulator of eosinophil generation, activation, recruitment, and survival, IL-5 contributes to eosinophilic infiltration in tissues (17, 18). Therefore, by targeting IL-5 (e.g., mepolizumab, depemokimab) or its receptor (e.g., benralizumab), the function of eosinophils, a key effector cell, can be directly attenuated, thereby controlling inflammation.

#### 2.1.3 Epithelial-derived alarmins (TSLP, IL-33, IL-25)

When stimulated by allergens, viruses, bacteria, and other external factors, nasal mucosal epithelium releases a group of cytokines known as “alarmins,” including thymic stromal lymphopoietin (TSLP), IL-33, and IL-25 (19). These alarmins are situated at the very top of the type 2 inflammatory cascade and can activate various immune cells (especially ILC2s), thereby initiating and amplifying the entire type 2 inflammatory response (20). Due to its broad downstream effects, TSLP is considered a “master regulator.” Targeting TSLP (e.g., tezepelumab) can block the inflammatory response at its source.

#### 2.1.4 The IgE pathway

CRSwNP patients, especially those with allergies, exhibit elevated IgE levels both systemically and locally in polyps. This IgE can bind to and activate mast cells and basophils, causing them to release histamine, leukotrienes, and other inflammatory mediators, further exacerbating the inflammatory response and tissue edema. Therefore, targeting IgE (e.g., omalizumab) provides another effective pathway for controlling CRSwNP, particularly in patients with comorbid allergic diseases (21).

### 2.2 Synergistic action of key immune and structural cells

The pathological process of CRSwNP is the result of the synergistic action of multiple cell types, forming a complex, self-amplifying inflammatory microenvironment.

#### 2.2.1 Eosinophils

Eosinophils have long been considered the core effector cells in CRSwNP. The granular contents they release, such as cationic proteins and peroxidases, are cytotoxic and can directly damage epithelial cells, leading to tissue destruction. However, recent studies using techniques like single-cell RNA sequencing have revealed a high degree of heterogeneity among eosinophils in polyp tissue, suggesting they may play more diverse roles than previously known (22). Furthermore, eosinophil-mediated inflammation drives olfactory dysfunction and neuroinflammation in animal models (23).

#### 2.2.2 B lymphocytes

While CRSwNP has traditionally been viewed as a disease driven primarily by T cells and eosinophils, growing evidence suggests that B lymphocytes and local antibody production also play a significant role (17). Polyp tissue contains a large number of B cells, which can form germinal center-like structures and produce various antibodies, including IgE. These findings challenge the traditional view and suggest that B cells may be another key element driving the chronicity and persistence of the disease (17).

#### 2.2.3 Macrophages and fibroblasts

Evidence suggests that ALOX15^+^ M2 macrophages, which are uniquely elevated in eosinophilic Chronic Rhinosinusitis with Nasal Polyps (eCRSwNP), play a pivotal role in pathological epithelial remodeling, with their production of CCL13 being a key mechanistic link (24). Fibroblasts in CRSwNP are no longer viewed merely as cells responsible for extracellular matrix(ECM) deposition and fibrosis, but also as active participants that critically trigger and amplify inflammation (25). Cytokines like IL-1β can also induce the transdifferentiation of epithelial cells and fibroblasts, a process known as Epithelial-Mesenchymal Transition (EMT), which not only promotes neutrophil recruitment but is also closely related to the formation and growth of polyps (4, 26).

### 2.3 Other inflammatory patterns

Although type 2 inflammation is the predominant endotype in CRSwNP, not all patients follow this pattern. Some patients, particularly in Asian populations, may exhibit non-type 2 or mixed inflammatory features, where neutrophils may play a more significant role (26–28). Additionally, dysbiosis of the nasal microbiota is thought to participate in the development of CRSwNP by affecting the local immune response (29). These non-classical inflammatory patterns explain poor response to some therapies and highlight disease heterogeneity and future personalized treatment complexity.

#### 2.3.1 GZMK^+^ CD8^+^ T cells and the complement pathway: a new mechanism driving disease recurrence

A recent breakthrough study has uncovered a novel mechanism for CRSwNP recurrence that is independent of the classic type 2 inflammatory pathway. The study found that in recurrent nasal polyp tissue, there exists a persistent population of memory CD8^+^ T cells that express Granzyme K (GZMK). These cells secrete GZMK to cleave and activate various components of the complement system, including C3 and C5, thereby driving a non-type 2 pro-inflammatory response. This discovery provides a new perspective on understanding the stubbornness and high recurrence rate of the disease. More importantly, the study confirmed that tissue levels of GZMK are a more accurate predictor of disease severity and recurrence risk than traditional biomarkers such as eosinophil count or IL-5 levels. In animal models, inhibiting GZMK activity through pharmacological or genetic means effectively alleviated airway inflammation, suggesting that GZMK could be a highly promising new target for treating refractory, recurrent CRSwNP (14).

#### 2.3.2 Neuroimmune signaling

An emerging field of research has revealed a close “dialogue” between the nervous and immune systems in CRSwNP (30). Neurotransmitters modulate immune cells, and cytokines released by these cells in turn affect neuronal activity; this bidirectional neuro-immune signaling pathway contributes to both the generation of nasal symptoms such as pain and itching and the persistence of inflammation, offering a new perspective for understanding disease mechanisms and developing novel therapeutic targets (30).

**Figure 2:**
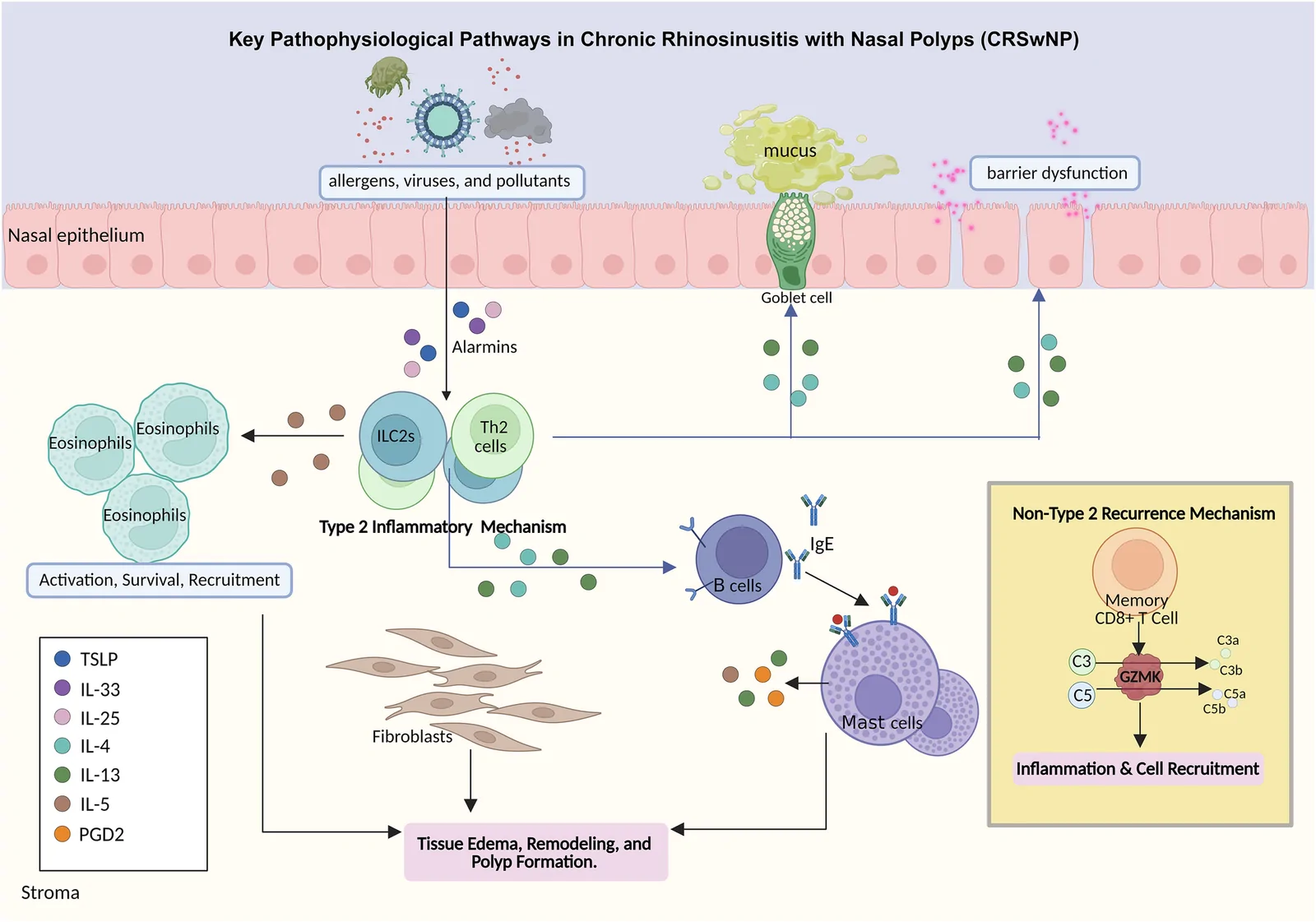
Key pathophysiological pathways in chronic rhinosinusitis with nasal polyps (CRSwNP). After stimulation by exogenous triggers such as allergens, viruses, and pollutants, the nasal mucosal epithelium releases alarmins (including TSLP, IL-33, and IL-25), initiating a type 2 inflammatory cascade. These alarmins activate type 2 innate lymphoid cells (ILC2s) and T helper 2 cells (Th2). IL-4/IL-13 secreted by these cells induces B cells to produce IgE, disrupts the epithelial barrier, and promotes mucus hypersecretion. IgE further activates mast cells to release inflammatory mediators, while IL-5 mediates the activation, survival, and recruitment of eosinophils. The combined effects of these immune cells and inflammatory factors on the mucosal stroma ultimately lead to tissue edema, remodeling, and polyp formation. In addition, the independent non-type 2 recurrence pathway labeled in the bottom right corner of the figure shows that granzyme K (GZMK) secreted by memory CD8⁺ T cells can directly induce inflammatory responses and immune cell recruitment by cleaving complement proteins C3 and C5, serving as a key parallel regulatory mechanism driving disease recurrence. Created with BioRender.com.

Evidently, the pathological mechanism of CRSwNP is a complex network (Figure 2). Therefore, intervening in upstream “master pathways” like TSLP or IL-4/IL-13 can deliver more comprehensive clinical benefits than targeting downstream eosinophils. This directly explains the outstanding outcomes of IL-4Rα and TSLP inhibitors across multiple endpoints and guides future development to focus on disrupting the network's most efficient strategic nodes for synergistic effects and multi-faceted benefits.

## 3 The traditional treatment ladder and its limitations in CRSwNP

Before the advent of biologics, the treatment strategy for CRSwNP primarily revolved around two pillars, medication and surgery.

### 3.1 Intranasal corticosteroids

Intranasal corticosteroids (INCS) are the cornerstone of medical therapy for CRSwNP (1, 31). Through their broad anti-inflammatory effects, they can reduce mucosal edema, shrink polyp size, and improve nasal symptoms. Non-aqueous formulations (e.g., nebulized, aerosol) offer a safe OCS-alternative without systemic side effects, while for aqueous formulations, long-term adherence is critical for achieving symptom control (32).

### 3.2 Functional endoscopic Sinus surgery (FESS)

For patients who do not respond well to or cannot tolerate medical therapy, FESS is the standard treatment option (11). The main goals of surgery include the complete removal of nasal polyps, opening and widening the ostia of all affected sinuses, and restoring ventilation and drainage functions of the nasal and sinus cavities. By clearing physical obstructions and inflammatory secretions, FESS can significantly alleviate symptoms and create favorable conditions for postoperative topical medication. Some modified or extended procedures, such as the Draf III frontal sinusotomy, are used to manage refractory cases involving all sinuses (33).

### 3.3 Unmet needs of traditional treatment

Although intranasal corticosteroids (INCS) and FESS are important for chronic rhinosinusitis with nasal polyps (CRSwNP), their limitations stand out, particularly high postoperative recurrence. Multiple long-term follow-up studies show that many patients experience polyp recurrence within years after surgery, requiring repeated systemic corticosteroids, biologic therapy or revision surgery (12, 34).

Clinical follow-up studies suggest that comorbid asthma, extensive sinus involvement on imaging and peripheral blood eosinophilia are important predictors of recurrence and revision surgery need (12). A 5-year cohort study of 130 patients after endoscopic sinus surgery reported a recurrence rate of 35.4%, with 17.7% of patients requiring revision surgery; comorbid asthma, frontal sinus involvement, higher Lund-Mackay CT score, higher endoscopic nasal polyp score and peripheral blood eosinophilia were associated with worse postoperative prognosis (12, 34). This is clinically crucial: these so-called “surgical failure predictors” are actually biomarkers of persistently active inflammatory endotypes. In short, postoperative recurrence results from uncontrolled, persistent underlying immune dysregulation. Recent molecular evidence further shows that memory CD8^+^ T cells expressing granzyme K (GZMK) exist in recurrent polyp tissue and drive proinflammatory responses independent of type 2 inflammation by activating the complement system, becoming a major driver of recurrence (14).

This changes our understanding of patients with postoperative recurrence. Previously, clinicians often chose “more potent drugs” or “more extensive revision surgery”. Now, recurrence is seen as an important diagnostic clue, indicating need for systemic, endotype-driven treatment. This turns the linear “medication→surgery→revision surgery” pathway into a more dynamic, individualized decision-making process. For patients at high recurrence risk, early biologic intervention even before initial surgery is a reasonable new clinical strategy.

## 4 Biologics: ushering in a new era of personalized treatment for CRSwNP

Biologics have revolutionized treatment for refractory CRSwNP by precisely targeting core inflammatory pathways.

A detailed summary of biologic agents for CRSwNP is provided in the Table 1.

**Table 1:** Overview of major biologics for chronic rhinosinusitis with nasal polyps (CRSwNP).

Drug class	Representative drug	Target	Key clinical trials
Anti-IL-4Rα Agents	Dupilumab	IL-4 receptor α subunit	SINUS-24, SINUS-52
	Stapokibart	IL-4 receptor α subunit	CROWNS-2 (phase III; JAMA 2025)
Anti-IL-5/IL-5R agents	Mepolizumab	IL-5	SYNAPSE
	Depemokimab	IL-5	ANCHOR-1, ANCHOR-2 (41)
	Benralizumab	IL-5 receptor α subunit	Phase III randomized trial (42); ORCHID (43)
Anti-IgE Agents	Omalizumab	Circulating IgE	POLYP 1, POLYP 2 (18)
Anti-TSLP Agents	Tezepelumab	TSLP	WAYPOINT
Anti-IL-33 Agents	Itepekimab	IL-33	CEREN-1 (60), CEREN-2 (61); phase II CRSsNP trial (59, 60)

### 4.1 Targets and clinical evidence of Major biologics

#### 4.1.1 Anti-IL-4R agents (dupilumab, stapokibart)

Dupilumab, a monoclonal antibody targeting the shared receptor subunit IL-4Rα for IL-4 and IL-13, exerts dual inhibition of two central type 2 inflammatory pathways. Its well-established efficacy and safety profile in chronic rhinosinusitis with nasal polyps (CRSwNP) is supported by robust clinical evidence, including the pivotal SINUS-24 and SINUS-52 trials which demonstrated significant improvements in nasal polyp score (NPS), patient-reported outcomes (SNOT-22), and olfactory function (35). Long-term real-world studies spanning up to 3 years, have confirmed the sustained therapeutic effect without significant attenuation (36, 37).

Furthermore, dupilumab demonstrates synergistic benefits in CRSwNP patients with comorbid asthma, effectively addressing both upper and lower airway pathology. Stapokibart (CM310), another IL-4Rα-targeting monoclonal antibody, has now been supported by the randomized phase III CROWNS-2 trial in China. In this trial, 180 adults with severe uncontrolled CRSwNP were enrolled across 51 Chinese centers and received stapokibart 300 mg subcutaneously every 2 weeks or placebo for 24 weeks, both as add-on therapy to daily mometasone furoate nasal spray. Compared with placebo, stapokibart significantly reduced nasal polyp score and nasal congestion score at week 24, with consistent efficacy in both the overall population and patients with eosinophilic CRSwNP; CT scores, olfactory function and quality-of-life outcomes also improved, with an acceptable safety profile (38). These data provide important phase III evidence for a China-developed IL-4Rα biologic and strengthen the role of IL-4Rα blockade in CRSwNP, particularly in Asian populations.

#### 4.1.2 Anti-IL-5/IL-5R agents (mepolizumab, depemokimab, benralizumab)

Anti-IL-5/IL-5R agents (mepolizumab, depemokimab) exert their therapeutic effects by targeting the IL-5 pathway to suppress eosinophilic inflammation. The pivotal SYNAPSE study demonstrated that mepolizumab significantly reduces nasal polyp size, improves clinical symptoms, and decreases the need for revision surgery in patients with severe CRSwNP (39). Notably, a follow- up investigation revealed sustained therapeutic efficacy for up to 24 weeks after drug discontinuation (40), suggesting potential disease-modifying properties. The next-generation long- acting agent depemokimab, administered biannually based on results from the ANCHOR-1 and ANCHOR-2 trials, provides enhanced treatment convenience while maintaining effective disease control in CRSwNP patients (41).

Benralizumab, a humanized monoclonal antibody targeting the IL-5 receptor α subunit (IL-5Rα), exerts its therapeutic effect through antibody-dependent cell-mediated cytotoxicity (ADCC) to directly deplete eosinophils. A Phase III randomized controlled trial demonstrated that in patients with severe CRSwNP previously treated with systemic corticosteroids and/or FESS, benralizumab significantly reduced both nasal polyp score (NPS) and nasal congestion score (NCS) compared with placebo (42). Additional Phase III studies are evaluating its long-term efficacy and safety in CRSwNP (43).

#### 4.1.3 Anti-IgE agents (omalizumab)

Omalizumab exerts its therapeutic effect by binding to circulating IgE and preventing its attachment to effector cell receptors, thereby inhibiting allergic inflammation. Its efficacy in CRSwNP has been established through the POLYP 1 and POLYP 2 clinical trials (21), demonstrating particular suitability for patients with comorbid allergic asthma and a clear allergic predisposition (21).

#### 4.1.4 Anti-TSLP agents (tezepelumab)

**Figure 3:**
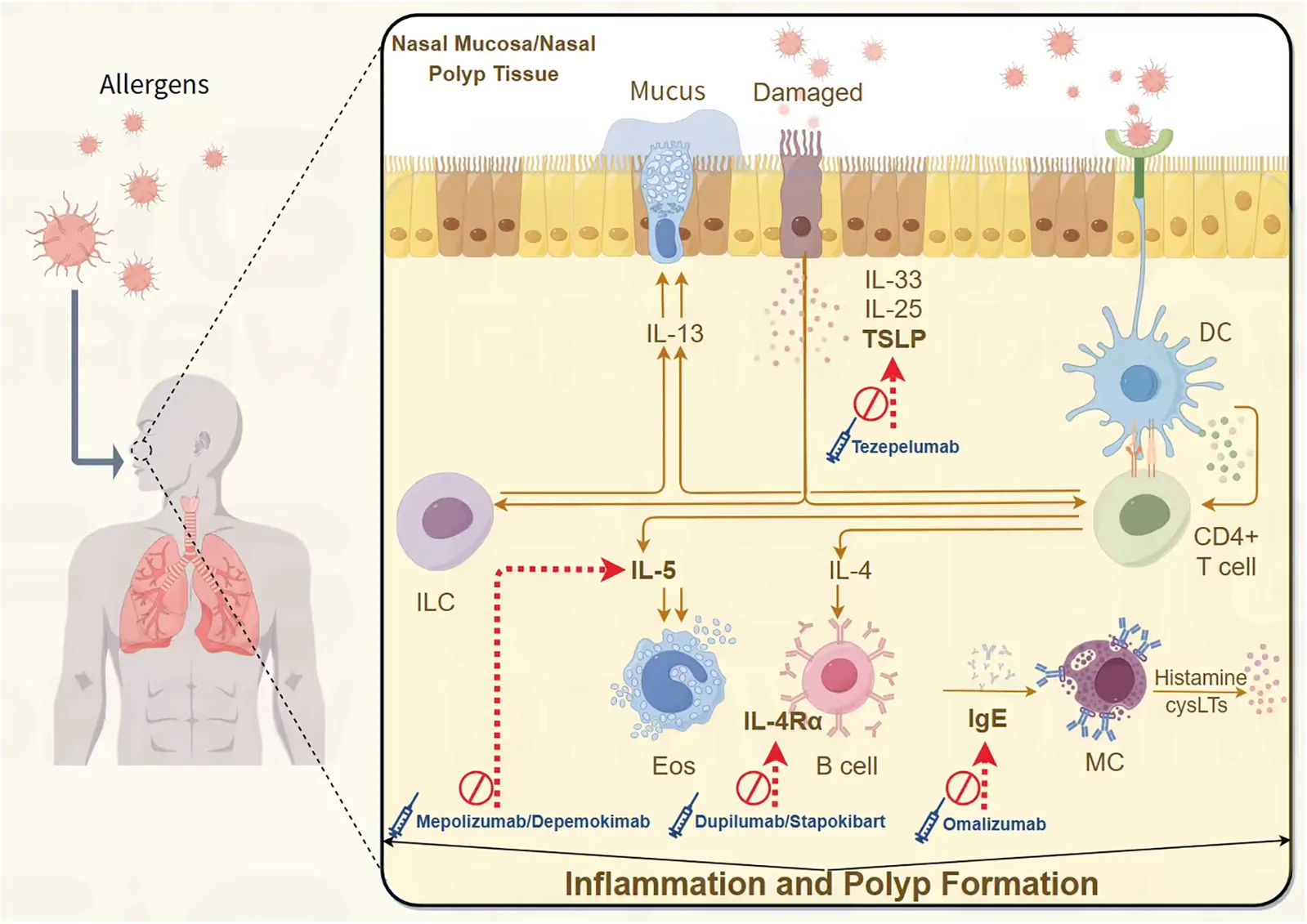
Inflammation and polyp formation. This schematic illustrates the pathological process of CRSwNP and the mechanism of action of biologic therapies. The left panel depicts a human silhouette, schematically representing the entry of allergens through the upper respiratory tract. The right panel shows a magnified view of the nasal mucosa/nasal polyp tissue, revealing the complete inflammatory cascade: allergens induce epithelial cells to release alarmins (TSLP, IL-33, IL-25), which in turn activate immune cells such as type 2 innate lymphoid cells (ILC2s) and T-helper cells, promoting the production of key cytokines (IL-4, IL-13, IL-5). These cytokines subsequently trigger a series of pathological events, including eosinophil (Eos) infiltration, IgE production by B cells, and mast cell (MC) activation, ultimately leading to tissue inflammation and polyp formation. The figure clearly marks the precise targets of various biologic agents with blue syringe icons. These include: Tezepelumab, which targets the upstream alarmin TSLP for broad intervention at the inflammatory source; anti-IL-4Rα drugs (Dupilumab, Stapokibart) that achieve dual inhibition of central type 2 inflammatory pathways by blocking the shared IL-4Rα subunit of the IL-4 and IL-13 receptors; anti-IL-5/IL-5R drugs (Mepolizumab, Depemokimab) that suppress eosinophilic inflammation by targeting the IL-5 signaling pathway; and Omalizumab, which binds to free IgE, preventing its interaction with Fc*ε*RI receptors on mast cells and basophils, making it particularly suitable for patients with allergic characteristics. Created with BioRender.com.

Tezepelumab exerts its therapeutic effect by targeting thymic stromal lymphopoietin (TSLP), an alarmin positioned upstream in the inflammatory cascade. By inhibiting inflammatory signaling at its source, this mechanism theoretically enables suppression of a broader spectrum of downstream pathways. The phase III WAYPOINT trial demonstrated significant efficacy in severe CRSwNP patients, with notable improvements in nasal polyp scores and congestion symptoms (44). Due to the broad nature of its mechanism of action, tezepelumab may also be effective for patients with atypical inflammatory features. Its exact place in the treatment of CRSwNP awaits further research (45) (Figure 3).

### 4.2 Efficacy comparison and selection considerations for different biologics

The selection of biologics for CRSwNP requires careful consideration due to the absence of direct comparative trials. Current evidence relies on indirect comparisons and real-world data (45, 46). Treatment selection should be guided by patient-specific characteristics: anti-IgE or anti-IL-4Rα therapies may be preferred for patients with prominent allergic/asthmatic features (21, 47), anti-IL-5 agents may be considered for those with eosinophil-predominant disease (39), and anti-TSLP therapy may be suitable for complex or non-type 2 inflammation (44). Individual treatment goals such as olfactory improvement should also inform decision-making (48, 49). Ultimately, optimal management requires “biologic stewardship”—personalized treatment selection based on thorough understanding of both patient endotype and drug characteristics.

### 4.3 Impact of biologics on key clinical endpoints

Biologics demonstrate significant benefits across key clinical domains in CRSwNP management. Regarding olfactory function—one of the most quality-of-life-impacting symptoms—these agents, particularly dupilumab, achieve substantial smell restoration (48, 49) through profound suppression of type 2 inflammation in the olfactory neuroepithelium. In comorbidity management, biologics exemplify the unified airway approach, producing dual benefits in patients with comorbid asthma by concurrently improving upper and lower respiratory outcomes through targeted pathway inhibition (10, 47).

#### 4.3.1 Surgical need and imaging changes

Biologics significantly reduce dependence on oral corticosteroids and the need for revision surgery (34, 50), mitigating long-term steroid side effects and reducing patient burden. Post-treatment CT imaging objectively demonstrates resolution of sinus inflammation, providing radiological confirmation of treatment efficacy (51). However, despite these clinical benefits, the real-world application of biologics faces significant health economic challenges owing to high drug acquisition costs (52, 53). Accumulating real-world evidence indicates that these direct medical costs can be partially offset by marked reductions in subsequent healthcare resource utilization, including fewer courses of systemic corticosteroids and reduced revision surgery rates (54, 55).

### 4.4 Emerging therapeutic targets and next-generation biologics

#### 4.4.1 Anti-IL-33 agents (itepekimab)

Epithelial-derived alarmin interleukin-33 (IL-33) lies upstream of the type 2 inflammatory cascade. It activates type 2 innate lymphoid cells and Th2 cells to drive eosinophilic inflammation and may indirectly participate in mixed inflammatory responses (19, 20, 56). Because conventional biologics targeting IL-4Rα, IL-5 or IgE mainly block downstream type 2 pathways, IL-33 blockade may provide a complementary strategy for patients with mixed eosinophilic-neutrophilic inflammatory features. Itepekimab is the most clinically advanced anti-IL-33 monoclonal antibody. In moderate-to-severe asthma, phase II data showed that itepekimab reduced asthma exacerbation risk and improved lung function, while COPD data suggested that benefit was concentrated in selected airway inflammatory phenotypes rather than neutrophil-dominant disease alone (57, 58). In CRSwNP, complete phase III endpoint data have not yet been reported. Two global phase III trials, CEREN-1 (NCT06834347) and CEREN-2 (NCT06834360), are evaluating itepekimab in inadequately controlled CRSwNP; the NCT06691113 phase II proof-of-concept trial is also exploring its use in chronic rhinosinusitis without nasal polyps (59–61). These studies will clarify whether upstream IL-33 inhibition can improve outcomes in refractory or mixed-endotype CRS populations.

#### 4.4.2 Targeting GZMK: a novel strategy for recurrent CRSwNP

Recent studies have identified granzyme K (GZMK)-expressing memory CD8⁺ T cells as a novel driver of CRSwNP recurrence independent of classical type 2 inflammation (14). GZMK promotes complement activation and sustains chronic airway inflammation, while tissue GZMK levels correlate more closely with disease severity and recurrence than conventional biomarkers (14). Although no GZMK-targeted biologics have entered clinical development, experimental inhibition of GZMK using the serine protease inhibitor d-Phe-Pro-Arg-chloromethylketone (PPACK), together with genetic deletion approaches, significantly alleviated airway inflammation in preclinical models, supporting GZMK as a promising therapeutic target for refractory or recurrent CRSwNP (14).

#### 4.4.3 OX40/OX40L pathway inhibition

The OX40/OX40L co-stimulatory pathway contributes to the maintenance of type 2 inflammation by promoting T-cell activation and survival. Increased OX40L expression has been observed in eosinophilic CRSwNP and is positively associated with tissue eosinophilia, suggesting its involvement in disease pathogenesis (62). Several monoclonal antibodies targeting OX40 or OX40L, including rocatinlimab and amlitelimab, have demonstrated promising efficacy in atopic dermatitis and are being evaluated in other type 2 inflammatory diseases (63, 64). Although clinical evidence in CRSwNP is currently lacking, modulation of the OX40/OX40L axis may represent a potential future therapeutic strategy by suppressing upstream T-cell activation.

## 5 Clinical management strategies and future outlook in the era of biologics

The adoption of biologics necessitates evolution in clinical management models, presenting new challenges for optimal treatment implementation.

### 5.1 Guideline consensus and patient selection

The European Position Paper on Rhinosinusitis and Nasal Polyps (EPOS 2020) and the EUFOREA statement indicate that biological agents may be used for patients with chronic rhinosinusitis with nasal polyps (CRSwNP) whose condition remains uncontrolled despite appropriate medical treatment and sinus surgery, and who meet 3 out of the 5 following criteria: presence of Type 2 inflammation, regular need for systemic corticosteroids or presence of contraindications to their use, significant impact on quality of life, loss of smell, and comorbid asthma (2, 65, 66).

Before starting biologic therapy for CRSwNP, a comprehensive assessment is essential—including detailed medical history, nasal endoscopy, sinus CT, possible allergic status and peripheral blood eosinophil count (10). In some cases, polyp pathological analysis may also help identify inflammatory endotypes (67). While guideline criteria vary slightly, their core is consistent: selecting suitable regimens for refractory patients in greatest need (68).

### 5.2 Efficacy assessment, definition of remission, and treatment switching

#### 5.2.1 Outcome measures

Biologic therapy is a long-term process, so it is essential to use standardized tools for regular efficacy assessment. International consensus recommends a multidimensional assessment system that includes patient-reported outcomes (such as SNOT-22), clinician-assessed measures (such as NPS), and objective measures (such as olfactory tests) (69, 70).

#### 5.2.2 Defining remission

as biologics deliver significant therapeutic effects, the treatment goal for CRSwNP shifts from “symptom improvement” to “remission”—a prolonged state of control, without bothersome symptoms for at least 12 months, no oral corticosteroids or FESS, no endoscopic active disease signs, and usually bilateral NPS ≤1, though its exact criteria remain to be explored (71).

#### 5.2.3 Treatment switching

Switching between biologics of the same class has limited effectiveness, while switching across different mechanisms of action is more clinically valuable, which can provide guidance for the clinical use of biologics. A Canadian multicenter study showed that mepolizumab and omalizumab were mostly switched (to dupilumab) due to insufficient efficacy (72). This may be attributed to differences in their mechanisms of action: dupilumab targets interleukin (IL)-4, which acts more upstream in the inflammatory cascade than anti-IL-5 or anti-immunoglobulin E (IgE) agents.

### 5.3 Summary and future directions

#### 5.3.1 Summary

The emergence of biologics has brought new hope to patients with type 2 inflammatory CRSwNP. The clear ability of biologic therapy to achieve individualized treatment has greatly promoted the development of precision medicine (13, 73, 74). However, the success of biologics has also brought a series of new problems. These problems have mapped out the future research directions and require doctors to possess new knowledge and skills to deal with the long-term management of chronic diseases.

#### 5.3.2 Future research

Currently, there is a lack of validated biomarkers (from blood, nasal secretions or tissues) to predict CRSwNP patients’ response to specific biologics, an urgent need for individualized treatment (4, 67) 13, 66. Though “≥10 eosinophils/HPF” screens biologic candidates, tissue GZMK—with better prediction for severe nasal polyps than traditional indicators (14)—needs further exploration. Also, improve use of existing biomarkers (tissue eosinophil count, Charcot-Leyden crystals) and solve difficult periostin detection.

Head-to-Head Clinical Trials: Well-designed, randomized controlled trials directly comparing different biologics are urgently needed to resolve uncertainties from current indirect comparisons and provide stronger evidence for clinical decisions.

Long-term Effects and Disease Modification: Longer follow-up studies are needed to assess biologics’ long-term safety, their potential “disease-modifying” effect on the disease's natural course, and optimal treatment duration.

Treatment of Non-Type 2 CRSwNP:Current anti-type 2 biologics have limited efficacy in patients with non-type 2 or mixed inflammatory endotypes of CRSwNP. Thus, developing new targeted therapies (e.g., targeting neutrophil-mediated inflammatory pathways) is a key future research direction (27, 28). Notably, a newly identified complement activation pathway driven by GZMK-expressing CD8^+^ T cells offers a theoretical basis for novel targeted drugs (e.g., GZMK inhibitors) in patients with recurrent or non-type 2 inflammation-dominant CRSwNP.

Health Economics: Given biologics’ high cost, rigorous cost-effectiveness analyses and exploration of more economical dosing strategies (such as adjusting intervals by disease activity) are crucial to ensure accessibility of these therapies and healthcare system sustainability.

## Data Availability

The original contributions presented in the study are included in the article/Supplementary Material, further inquiries can be directed to the corresponding authors.
